# *Pasteurella* Endocarditis: A Case Report and Statistical Analysis of the Literature

**DOI:** 10.1155/2020/8890211

**Published:** 2020-07-20

**Authors:** Randall S. Porter, Christine M. Hay

**Affiliations:** ^1^University of Rochester School of Medicine and Dentistry, 601 Elmwood Ave, Rochester, NY 14642, USA; ^2^Department of Medicine, Infectious Diseases Division, University of Rochester Medical Center, 601 Elmwood Ave, Rochester, NY 14642, USA

## Abstract

*Pasteurella* is a genus of commensal bacteria of the oral cavity of several domesticated animals and a common cause of cellulitis after animal bites. *Pasteurella* has also been reported as a rare cause of endocarditis, with only 35 prior cases of definite *Pasteurella* endocarditis in the literature. Here, we present a case of *Pasteurella multocida* endocarditis treated successfully with surgery and antibiosis, as well as a review of the literature with statistical analysis of correlations between risk factors and clinical outcomes, as well as between treatment choices and clinical outcomes. Despite the small sample size, our analysis indicates a statistically significant correlation between comorbid liver disease and mortality, as well as a significant negative correlation between surgical treatment and mortality. This analysis implies a need for surgical management of endocarditis due to *Pasteurella* species and for more aggressive management of *Pasteurella* endocarditis in the setting of comorbid liver disease.

## 1. Introduction

*Pasteurella* is a genus of Gram-negative, facultative anaerobic, nonmotile bacilli which often exhibit pleomorphism and bipolar staining on microscopy. As indigenous flora of the oral cavity of many domesticated and tamed animals, including common pets and farm animals, they can be a relatively common cause of cellulitis after bites and scratches obtained from handling such animals. Endocarditis is a rare manifestation of *Pasteurella* infection and is typically due to *Pasteurella multocida*, often following known animal exposure. Only 35 cases of definite *Pasteurella* endocarditis have been reported prior to our case in the literature, primarily due to *Pasteurella multocida*. Due to its rarity and relatively high mortality, ongoing reporting of novel cases and analysis of the literature is vital to determining appropriate management of *Pasteurella* endocarditis.

## 2. Case

Our patient is a 66-year-old Caucasian woman with a complicated medical history of third-degree atrioventricular block (3° AV block) status post implantation of a Boston Scientific dual chamber internal cardioverter-defibrillator (ICD) with subpectoral pocket placement in Sep 2017, upgraded to dual-chamber ICD with right ventricular (RV) pacing lead extraction in Jan 2018, nonobstructive coronary artery disease (CAD) (50% mid-left anterior descending artery (LAD) stenosis; 20% mid-right coronary artery (RCA) stenosis), ventricular tachycardia, severe aortic valve (AV) stenosis status post minimally-invasive #21 On-X mechanical aortic valve replacement (AVR) in Sep 2017, thoracic aortic mycotic pseudoaneurysm status post repair in Oct 2017, hypertension, heart failure with preserved ejection fraction (HFpEF), chronic obstructive pulmonary disease (COPD) requiring 3 L/min O_2_, mild chronic normocytic anemia, chronic anxiety, and chronic depression. On Jul 23, 2019, the patient presented to our hospital with a 2-day history of decreased oral (PO) intake, myalgias, nausea, bilious vomiting, night sweats, chills, diarrhea, lightheadedness, stool incontinence, and headache, as well as a 1-day history of chest pressure. Initial examination was positive for ventricular tachycardia, ill appearance, loud S1, and jugular venous distention (JVD). Pertinent findings in social history included a pet cat that sleeps in her bed at night but no known scratches or bites, a former smoking history of 50 pack-years, 1-2 cans of beer per night, and no illicit substance use. Initial labs indicated hyponatremia of 129 mEq/L, a white blood cell count (WBC) of 11.8 cells/mm^3^, negative serum troponins, a serum lactate of 2.4 U/L, and a subtherapeutic international normalized ratio (INR) of 1.7. Chest X-ray (CXR) and computed tomography angiography (CTA) of the chest were negative for pathologic findings. She was started on an enoxaparin to warfarin bridge with a goal INR of 2-3, given intravenous (IV) fluid boluses, and had blood cultures drawn. On Jul 24, she was started on IV piperacillin-tazobactam 3.375 g every 8 hours (q8h). On Jul 26, she had increased work of breathing requiring bilevel positive airway pressure (BiPAP) for a few hours, and blood culture results indicated *Pasteurella multocida*, *Staphylococcus epidermidis*, and *Staphylococcus warneri* bacteremia. A transthoracic echocardiogram (TTE) was positive only for left atrial (LA) and left ventricular (LV) hypertrophy, aortic stenosis without regurgitation, moderate mitral regurgitation, mildly dilated ascending aorta, and moderately elevated pulmonary artery pressure, but no vegetations. On Jul 28, her piperacillin-tazobactam was discontinued and she was started on PO moxifloxacin 400 mg daily. She was discharged on Jul 30 with continued moxifloxacin, the last dose of which was planned for Aug 6 to complete a total of 14 days of antibiotic treatment. The patient improved as an outpatient until the end of her antibiotic course, and an outpatient visit on Aug 19 revealed only occasional chills and fatigue, which resolved later that month. By Sep 9, however, she had developed aortic valve insufficiency, and on Sep 28, she developed shaking chills and altered mental status (AMS). On Sep 30, she presented to her primary care provider (PCP), who sent her to our emergency department (ED) for suspected sepsis.

On presentation to the ED, the patient displayed AMS, dyspnea with orthopnea, headache, and rigors, and was febrile to 38.5°C, hypotensive at 80/57 mmHg, tachycardic at 114 BPM, and hypoxic to 75% oxygen saturation (SpO_2_) on room air. Her CXR showed bilateral pulmonary edema, and labs were notable for a brain natriuretic peptide (BNP) of 12,000 pg/mL, lactate of 3.8 U/L, and serum Na of 123 mEq/L. In the ED, she was placed on IV piperacillin-tazobactam 4.5 g, decreased to 3.375 g q6h after the first dose, IV azithromycin 500 mg daily, IV doxycycline 100 mg once, and IV linezolid 600 mg once, as well as BiPAP with a positive end-expiratory pressure (PEEP) of 6 cm H_2_O until her shortness of breath (SOB) improved. Azithromycin was discontinued on Oct 1 when blood cultures drawn on Sep 30 indicated Gram-negative bacteremia. Transesophageal echocardiogram (TEE) on Oct 2 indicated an aortic valve vegetation, as well as a paravalvular aneurysm on the aortic-mitral septum. By Oct 3, her bacteremia was identified as *Pasteurella multocida*. Repeat cultures were negative, and the patient clinically improved; she was discharged on Oct 8 with a continued course of IV piperacillin-tazobactam to be taken at home until Nov 5. She continued to improve, and at an outpatient visit on Oct 21, she reported no chills, fever, chest pain, chest discomfort, or presyncope. At an outpatient visit on Oct 28, however, she was found to be severely dyspneic with an SpO_2_ of 81% on room air, with rales, wheezing, peripheral edema, and a diastolic murmur on exam. A TTE performed at that time indicated severe aortic regurgitation and a dilated right heart with severe tricuspid regurgitation, and she was readmitted.

On this admission, our patient described a gradual increase in SOB and home oxygen requirement, anorexia, and increased peripheral edema for 1 week, in addition to recent urinary hesitancy with oliguria. Exam indicated volume overload, and the patient was afebrile. Labs were notable for serum aspartate aminotransferase/alanine aminotransferase (AST/ALT) of 195/161 U/L, alkaline phosphatase of 208 U/L, and a normal WBC count. She was continued on piperacillin-tazobactam, home torsemide, and 3 L/min O_2_. Her condition improved, and she remained stable until surgery on Nov 7, which found prosthetic aortic valve endocarditis with an aortic root abscess extending into the RA and associated tricuspid annulus destruction. Bovine pericardium was used to repair the atrial perforation, cadaver allograft for a Bentall procedure, and a 33 Epic bioprosthetic valve for tricuspid replacement. Epicardial pacing wires were placed with the intent of replacement with dual-chamber ICD after resolution of her infection, and her chest was left open (due to difficulty mobilizing the chest wall) and packed and sealed with an iodine-impregnated incision drape (Ioban™). Postoperatively, she required epinephrine, milrinone, vasopressin, and mechanical ventilation. Her WBC and temperature waxed and waned between elevated and normal for several days postoperatively, and hypoxia and hypercapnia required an increasing fraction of inspired oxygen (FiO_2_). On Nov 9, she received a chest washout and primary chest closure. On Nov 10, initial culture from the tricuspid valve was positive for *Staphylococcus warneri* in addition to another organism later identified as a *Curtobacterium* sp.; in addition to her continued piperacillin-tazobactam, she was placed on IV daptomycin 8 mg/kg daily due to a questionable history of drug reaction with eosinophilia and systemic symptoms (DRESS) to vancomycin and contraindications (thrombocytopenia and anemia) to linezolid. She also was noted to have increasing abdominal distention at this time, and an abdominal CT showed dilated bowel with pneumobilia, which resolved over the next few days. On Nov 13, her *S. warneri* sensitivities indicated resistance to gentamicin and one colony to gentamicin and clindamycin but resistance in all colonies to cefazolin, erythromycin, linezolid, methicillin, oxacillin, penicillin G, tetracycline, and vancomycin; her daptomycin was discontinued at this time. She had return of bowel function on Nov 15. A possible infection of the right knee joint at this time resulted in the reinitiation of daptomycin for empiric methicillin-resistant *Staphylococcus aureus* (MRSA) coverage, discontinued on Nov 18 for lack of signs or symptoms concerning for joint infection. She was able to be weaned off vasopressor and inotrope support on Nov 16, and because of her development of ARDS and an expectation of prolonged intubation, she was given a tracheostomy and endotracheal tube on Nov 19. Her WBC had been slowly declining over this period, and by Nov 20, she was no longer leukocytotic.

On Nov 21, she had return of abdominal pain and worsening distension, both of which had previously been resolving, and an abdominal XR indicated evolving ileus. Abdominal CT on Nov 22 found pneumatosis intestinalis and significant free air concerning for bowel perforation, and she was given a single dose of metronidazole before emergent exploratory laparotomy and small bowel resection for distal ileal obstruction. Postoperatively, she required norepinephrine for 1 day. She developed worsening abdominal pain and distension on Nov 24, and blood cultures drawn on Nov 21 were found to be positive for Gram-negative rods later identified as *Klebsiella aerogenes*; ciprofloxacin 400 mg IV daily was started at this time, due to the patient's history of allergies to ceftriaxone and ertapenem. By Nov 27, return of bowel function was noted, repeat blood cultures had failed to grow any bacteria, and *Klebsiella* sensitivities indicated resistance to ampicillin, ampicillin-sulbactam, cefazolin, ceftriaxone, and piperacillin-tazobactam, dose-dependent susceptibility to cefepime, and sensitivity to trimethoprim-sulfamethoxizole, tobramycin, meropenem, amikacin, and most importantly, ciprofloxacin, her last dose of which was planned for Nov 30. However, her WBC elevated from 6.4 to 12.9 cells/mm^3^ overnight, and by Nov 28, it was at 14.7 cells/mm^3^ in addition to the development of abdominal distention and an abdominal XR indicating a dilated small bowel concerning for ileus and possible ischemia. A CT that day indicated free air and was concerning for an anastomotic leak; emergent surgery confirmed bowel contents in the peritoneum due to dehiscence of the anastomosis, which was resected prior to an ileostomy. Her piperacillin-tazobactam and ciprofloxacin were discontinued, and she was started on meropenem 2000 mg q8h.

On Nov 30, she became febrile and leukocytotic to 21.1 cells/mm^3^, and her antibiotic regimen was adjusted to meropenem 500 mg q6h, daptomycin 8 mg/kg daily, and fluconazole 400 mg daily. She was afebrile by the following day, but her WBC count lowered only slightly over the next few days, and her fever had returned by Dec 6. A CT on Dec 6 indicated two abdominal rim-enhancing loculated fluid collections concerning for abscesses. Her daptomycin was discontinued, and she received an abscess drainage of 9 mL of purulent fluid, drain placement, and paracentesis of 40 mL clear yellow to red fluid on Dec 7. She became afebrile, and her WBC decreased to 14.9 cells/mm^3^, which continued to decrease over Dec 8 into the normal range. On Dec 10, surgical pathology of the resected bowel anastomosis was found to be consistent with cytomegalovirus (CMV) enteritis, and she was started on ganciclovir 5 mg/kg q12h on Dec 11 after a positive CMV quantitative polymerase chain reaction (qPCR) of 9419 IU/mL. By Dec 11, her abscess fluid had also cultured *Enterococcus faecium*. Her meropenem was discontinued, and she was started on linezolid 600 mg q12h and piperacillin-tazobactam 4.5 g q8h. Her WBC count continued to decrease and by Dec 15, she was leukocytopenic to 3.8 cells/mm^3^. By Dec 17, her leukocytopenia had worsened to 2.8 cells/mm^3^, and her ganciclovir was discontinued in favor of foscarnet 3500 mg q12h. On Dec 19, her piperacillin-tazobactam was discontinued to complete her endocarditis treatment. An abdominal CT on Dec 23 showed complete resolution of her prior abscesses, and her linezolid was discontinued. Her abscess drains were removed on Dec 24, and her foscarnet was discontinued, as she had also completed her 14-day course of treatment for CMV enteritis.

Despite her recurrent infections, over the course of December our patient continued to slowly improve in respiratory and hemodynamic status. By Dec 22, she was ambulatory and could tolerate unassisted ventilation with tracheostomy trials of 3.5 hours, which improved rapidly over the next week to complete freedom from mechanical ventilation on Dec 28. On Dec 30, after over 48 hours off mechanical ventilation, she was given a Metronic automatic ICD, and her epicardial pacing wires were removed. By Jan 10, 2020, she was able to tolerate endotracheal tube capping of over 24 hours with good oxygenation, and her endotracheal tube was removed.

Our patient was discharged on Jan 13, 2020 to a skilled nursing facility (SNF) for rehabilitation. At the time of discharge, she had a well-functioning ileostomy requiring PO NaCl supplementation due to ileostomy losses, but otherwise enjoyed a regular diet. She was oxygenating well on room air after removal of her endotracheal tube, and her heart was well-paced by her ICD. After less than one week in her SNF, she was released home. In follow-up, she was seen by cardiothoracic surgery (Jan 23) and cardiology (Jan 29 and Feb 4). Over this time, paroxysmal atrial flutter was noticed in addition to one 15-second episode of ventricular tachycardia. On Feb 9, she was admitted to an out-of-network hospital for pneumonia which resolved after a short stay in the ICU; she was discharged home on Feb 18. She was seen again by cardiology on Mar 2 and cardiothoracic surgery on Mar 4 and showed improvement from her Feb 4 and Jan 29 cardiology appointments, with resolution of her atrial flutter. She had no further episodes of ventricular tachycardia, and her low appetite, low weight, fatigue, anxiety, and depression, which had worsened over the course of her most recent hospital stay, were all improving. As of Mar 30, 2020, she was continuing to improve and had no new health concerns.

## 3. Literature Review

### 3.1. Methods

We queried the PubMed database for all articles resulting from the search “Pasteurella” AND “endocarditis.” All results were manually reviewed for relevance to our topic. Cases without microbiologically proven *Pasteurella* infection were excluded, as were cases which did not meet Duke or modified Duke criteria for the diagnosis of endocarditis. Articles in other languages were translated into English before review. Articles which met these criteria were examined for reference to cases previously described in the literature, and any such referenced articles novel to our search were reviewed in the same manner. After this search was completed, all articles which did not contain a novel case or otherwise add information which could not be extracted from the primary case literature were excluded. Each remaining case was examined, and pertinent information was extracted for statistical analysis.

In extracting information into a format useful for statistical analyses, some generalizations were made. Species of *Pasteurella* were not assumed to be that stated by each article at face value, particularly with respect to non-*multocida* species. Our earliest case presented to the hospital in 1962 [1], and many cases occurred prior to the molecular identification of *Pasteurella* species. Though distinction of *Pasteurella* from related genera, and typically distinction of *P. multocida* from non-*multocida Pasteurella* species,was feasible prior to the advent of these methods (papers which did not adequately categorize their infectious agents as definitively belonging to the *Pasteurella* genus having been excluded from our analysis), advancements have shown that even with current molecular methods, proper identification of non-*multocida* species remains challenging [2–10]. Therefore, we decided to categorize species as *multocida* and non-*multocida*, as many of the papers regarding *P. dagmatis*, *P. pnemotropica*, *P. haemolytica*, and *P. ureae* simply do not have enough information to retroactively verify the species identifications made. Likewise, to allow for easier and more robust statistical analysis of different factors' influence on case outcome, case outcome was reduced to a binary: death due to *Pasteurella* endocarditis or cure of endocarditis (regardless of sequelae); different categories of comorbidities were consolidated from individual diagnoses to broad categories (specifically, liver disease, heart disease, and substance abuse, as these were the most common broad categories of comorbidities across the literature).

Statistical analysis was carried out using Minitab^Ⓡ^ 19. Each test was applied over all cases (including our own) which had adequate data regarding the hypothesis being questioned. We set the *α*-value of each test to 0.05 prior to any analysis. Of the data extracted, we compared patient sex with the case outcome, as well as the species of *Pasteurella* (*multocida* versus non-*multocida*) with the outcome, using Fisher's exact test, as the contingencies of the *χ*^2^ test of association were not met, and Fisher's exact test is preferable in cases of low expected cell-values [11, 12]. For comparison of the case outcome across comorbidities, *χ*^2^ contingencies were not met, and as we used three comorbidity categories (as previously discussed), a Fisher's exact test could not be applied to the data; therefore, while not ideal, we used the Fisher's exact test to test the association of each of the three comorbidity classes with case outcome individually. The association between surgical versus nonsurgical management and case outcome was evaluated using the Cochran–Mantel–Haenszel method to control for the potentially confounding variable of which valve was affected on the case outcome [13, 14]. Finally, to counteract the possibility of finding falsely significant findings due to data mining, our raw *p* values were corrected using the Benjamini–Hochberg method [15–17] at a (conservative) *q* value of 0.05.

### 3.2. Results

Including our current case, a total of 36 cases of adequately-verified *Pasteurella* endocarditis have been reported in the literature, a summary of which may be found in Table 1. Of these 36 cases, 24 were cases of *Pasteurella multocida* [18, 19, 21, 24, 25, 27, 30–35, 37–39, 41–43, 45, 47–49] and 12 were non-*multocida* species (reported as follows: *P. dagmatis* (4) [5, 20, 28, 36], *P. pneumotropica* (3) [22, 40, 44], *P. haemolytica* (3) [1, 26, 46], and *P. ureae* (2) [23, 29]; though as noted above, distinguishing between these species can be quite difficult, and thus, we do not take these species designations to be inherently accurate beyond their designation as non-*multocida Pasteurella* spp.). Males had a higher incidence, accounting for almost 2/3 of the cases. The mean and median age at presentation were 56 and 57, respectively, with a standard deviation of 17 years and a reasonably even distribution between ages 35 and 85. Only one case occurred at an age >85 [42] and only 3 cases at an age <35 [22, 25, 44]. Twenty-five cases (69%) involved recent exposure to an animal, most commonly cats (17) [5, 18–21, 28, 30–32, 39, 41–43, 45, 49] and dogs (8) [19, 24, 27, 31, 34, 35, 39, 45], but also sheep (1) [38], rodents (1) [44], and fish (1) [44]. Of these, only 7 had a known history of animal bite [18, 20, 21, 28, 41, 42, 49], 2 with a known history of animal scratch [28, 40], 2 with a known history of an animal licking a wound [5, 27], and 3 with other wounds or indications of a local infectious nidus in the setting of animal exposure [18, 19, 35]; the other 12 cases of animal exposure occurred without any indication of a skin or soft tissue source [2, 24, 30–32, 34, 38, 39, 43–45]. The number of reported cases has increased over every decade since the first recorded case. Of those countries represented more than once, Saudi Arabia has reported 2 cases [26, 38], Japan 4 [29, 34, 47, 48], France 8 [18, 21, 22, 24, 35, 37, 46], and the United States 13 [2, 5, 19, 20, 23, 27, 28, 31, 32, 36, 43, 44]. While many patients presented with a past medical history (PMH) significant for substance abuse (7, including ethanol and nicotine abuse) [21, 23, 28, 30, 37, 39, 49], liver disease (8) [18, 21, 25, 28, 30, 31, 35], or heart disease (17) [2, 5, 18, 20, 25, 27–29, 31, 32, 34, 36, 40, 41, 44, 45], other comorbidities were noted, though in 6 cases [19, 24, 26, 38, 46, 47], there was no significant PMH presented.

Systemic coinfections were reported only twice, once with *Burkholderia cepaciae* and *Candida* spp. in a cirrhotic French cat owner [18] and once with *Streptococcus salivarius* in a northwestern African with a history significant only for poor dentition and without known animal exposure [33]. While our patient presented initially with *Staphylococcus epidermidis* and *Staphylococcus warneri* coinfections, this was long before the patient began to show any signs, symptoms, or radiographic evidence of endocarditis, and these coinfections never resurfaced after initial antibiotic therapy. Resistance to antibiotics in *Pasteurella* strains was scarcely described across the literature. One study each reported resistance to penicillin [33] and piperacillin-tazobactam [48] in *P. multocida* and to bacitracin and triacetyloleandomycin in a non-*multocida* species [2]. Of the remaining studies, only three explicitly stated that no resistances were identified [23, 26, 29], and all other studies failed to discuss resistance, making an accurate antibiogram at this time impossible. All valves have been affected in the literature: 15 cases of native aortic valves [18, 19, 21, 24, 26, 27, 30, 31, 35, 37, 38, 43, 45, 46, 49], 4 prosthetic aortic valves (3 bioprostheses [5, 32, 36] and 1 mechanical prosthetic [41]), 10 native mitral valves [2, 18, 22, 25, 28, 29, 33, 34, 47, 48], 2 native tricuspid valves [42, 44], 1 native pulmonary valve [39], 1 dual-infection of native mitral and tricuspid valves [23], and our dual-infection of metal aortic prosthesis and native tricuspid valve. Complications and sequelae arose in most cases, which appeared primarily to be complications of sepsis and endocarditis or attributable to preexisting conditions, in many cases causing organ failure; only the reproductive system appeared spared from complications and sequelae across cases, though that may well be due to difficulty in acutely diagnosing unsuspected reproductive organ failure.

For treatment, all patients received antibiotics except for a patient who died shortly after presentation [21], typically with penicillins once culture and sensitivity results were available. Duration of antibiotic therapy varied widely both in cured patients and in those who died of their disease, often due to complications requiring elongation of the initially-intended duration of therapy, but also due to differences in the initial choice of antibiotic therapy. We considered analyzing the association between duration of antibiotic therapy and case outcome, but due to the varied reasons for elongation or shortening of the antibiotic therapy duration across cases, it was decided that the results would have little utility due to the difficulties inherent in the interpretation of any potential results. In 16 cases, valve replacement was utilized for source control of the infection [5, 19, 22, 24, 33–35, 38–41, 46–49]. In total, 7 cases resulted in the patient's death during the course of endocarditis treatment, all of which occurred in the absence of surgical source control, representing 19% of all cases and 35% of cases in which there was no surgical management of the affected valve [2, 18, 21, 25–27, 31].

### 3.3. Statistical Analysis

Fisher's exact test between patient sex and case outcome was conducted due to a noted difference in the death rate between the sexes; this difference was not statistically significant (*p* values: 0.4327 raw/0.8654 corrected). Species (*P. multocida* versus all other *Pasteurella* species studied as one group) was likewise not found to be significantly associated with case outcome (*p* values: 0.6910 raw/0.8292 corrected) using the same method. Individual Fisher's exact tests between the most prevalent comorbidities (liver disease, heart disease, and substance abuse) and case outcome resulted in *p* values of 0.0064 raw/0.0385 corrected, 0.4338 raw/0.6508 corrected, and 1.0000 raw/1.0000 corrected, respectively. This indicates that the observed increase in mortality in those with liver disease (62.5% vs. 10.7% in those without liver disease) was statistically significant, while heart disease and substance abuse were not significantly associated with case outcome at our level of statistical power. Note that the *p* value of the association between substance abuse and outcome is exactly 1 because the observed and expected values of each cell differed by <1. A Cochran–Mantel–Haenszel test of the association between surgical treatment and case outcome resulted in an odds ratio (OR) of dying after valve replacement compared to that with medical therapy alone of exactly 0 with a statistically significant *p* value of 0.0093 raw/0.0278 corrected. Recall that the odds ratio is exactly 0 because no patients who received a valve replacement in the literature died of their endocarditis. Corrected *p* values after Benjamini–Hochberg adjustment did not indicate any likely false discoveries at a conservative *q* value of 0.05. We initially intended to also test the association between the number of days from the onset of signs or symptoms and the surgical valve replacement with case outcome using logistic regression; however, since no patients treated surgically died in the course of treatment, the test is mathematically impossible to complete, leaving us with no information as to any possible connection between case outcome and the number of days between the onset of symptoms and valve replacement.

## 4. Discussion

Our case is notable as the only case in the literature to involve the aortic and tricuspid valves, only one of 2 cases involving multiple valves [23], and only one of 2 cases involving a mechanical prosthesis [41]. Our patient's recurrent course and multiple hospitalizations are likely due to seeding of her aortic prosthesis early in her first bout of sepsis, which then grew and was managed too conservatively in her second hospitalization. It took 110 days from the onset of symptoms to surgical intervention for source control of her endocarditis. Despite this prolonged clinical course, an extensive cardiac history, and multiple comorbidities, our case provides yet another example of the trend noted in the literature that all reported patients with *Pasteurella* endocarditis treated surgically have survived, while the overall mortality rate in reported patients without surgical intervention is 35%.

Considering that only 36 cases of definite *Pasteurella* endocarditis exist in the literature (including the case presented here), it was not expected that our statistical analyses would have enough power to detect statistically significant results; it is quite possible that as the number of cases in the literature grows, further analyses will detect more statistically significant associations which may be of benefit in understanding typical progression of the disease and the most appropriate management. Considering the overall reported mortality of 19%, it is impressive that none of the 16 patients on record who have received valve replacement surgery have died; likewise, the difference in mortality for those with comorbid liver disease, 62.5% versus 10.7% without liver disease, is striking. That these associations were found to be statistically significant even in such a small sample likely indicates a very strong correlation. While it has been vaguely suggested in the past that surgical source control is highly desirable in *Pasteurella* endocarditis, our results confirm this with strong statistical validity and suggest that (at least until further research can be conducted on appropriate management) valve replacement should be offered to all patients presenting with *Pasteurella endocarditis* who lack absolute contraindications to surgical intervention. It is unfortunate that the 100% survival after surgical intervention makes it mathematically impossible to evaluate any potential association between the duration of time between presentation to the hospital and valve replacement with case outcome; with the little data we have at present, we cannot determine when valve replacement should ideally be offered in the course of treating *Pasteurella* endocarditis, but simply that it should be offered. Considering the apparent success of valve replacement for source control across the literature, it is surprising that it was not offered to more than 44% of patients, though this is perhaps understandable given the multiple comorbidities, and thus risk factors for surgery, with which many of these patients presented. Still, it is our hope that the significance of our results leads to surgery being offered more consistently to *Pasteurella* endocarditis patients and, further, that patients with liver disease are watched especially closely for any sign of deterioration during the course of hospitalization for *Pasteurella* endocarditis. *Pasteurella* is a rare cause of endocarditis, and thus its ideal management has not yet been fully determined. Considering its high mortality risk, we must make full use of any significant associations we can glean from analysis of the literature, few though these associations may be at present.

## Figures and Tables

**Table 1:** Summary of the reviewed articles. The “Year” category lists the year of patient presentation, not of the associated publication; “Age” is likewise the patient's age at presentation. Note that due to varying and unreliable verification techniques, identifications of *Pasteurella* species other than *multocida* should be doubted beyond their identity as non-*multocida Pasteurella* spp. The “Wound” category can relate directly to the “Exposure” category (e.g., the 1998 Camou et al. [18] case is that of a cat bite to the calf) or be unrelated (e.g., the 2013 Mikaberidz et al. [19] case is that of a patient who lived with dogs and cats and had an unrelated sacral ulcer prior to presentation). “Cure” in the “Outcome” category is defined as complete resolution of all signs and symptoms of endocarditis.

First author	Year	Age	Sex	Location	*Pasteurella* species	Resistance	Coinfection	Valve	Exposure	Wound	Surgery	Outcome
Doty [2]	1962	54	F	USA	*Haemolytica*	Bacitracin, triacetyloleandomycin	None	Mitral	“Domestic pets”	None known	None	Death, presumed from *Pasteurella* endocarditis
Gump [20]	1972	44	M	USA	*dagmatis*	Unknown	None	Unknown	Cat	Bite, hand	None	Cure
Guerin [21]	1980	62	F	France	*multocida*	Unknown	None	Aortic	Cat	Bite, foot	None	Death, presumed from *Pasteurella* endocarditis
Cornaert [22]	1982	33	F	France	*pneumotropica*	Unknown	None	Mitral	Unknown	None known	Valve replacement	Cure
Brass [23]	1983	40	M	USA	*ureae*	None	None	Mitral, tricuspid	Unknown	None known	None	Coma
Salmon [24]	1986	63	F	France	*multocida*	Unknown	None	Aortic	Dog	None known	Valve replacement	Cure
Thamlikitkul [25]	1989	17	M	Thailand	*multocida*	Unknown	None	Mitral	None	None known	None	Death, presumed from *Pasteurella* endocarditis
Yaneza [26]	1990	40	M	Saudi Arabia	*haemolytica*	None	None	Aortic	None	None known	None	Death, presumed from *Pasteurella* endocarditis
Hombal [27]	1991	61	M	USA	*multocida*	Unknown	None	Aortic	Dog	Licked leg ulcers	None	Death, presumed from *Pasteurella* endocarditis
Sorbello [28]	1992	55	F	USA	*dagmatis*	Unknown	None	Mitral	Cat	Scratched, bitten	None	Cure
Yamamoto [29]	1992	59	M	Japan	*ureae*	None	None	Mitral	Unknown	None known	None	Cure
Genné [30]	1994	38	F	Switzerland	*multocida*	Unknown	None	Aortic	Cat	None known	None	Cure
Vasquez [31]	1997	65	M	USA	*multocida*	Unknown	None	Aortic	Dog, cat	None known	None	Death, presumed from *Pasteurella* endocarditis
Nettles [32]	1997	72	F	USA	*multocida*	Unknown	None	Aortic (prosthesis)	Cat	None known	None	Cure
Camou [18]	1998	79	F	France	*multocida*	Unknown	None	Mitral	Cat	Bite, calf	None	Death from acute heart failure
Abad [33]	1999	37	M	NW Africa	*multocida*	Penicillin	*Streptococcus salivarius*	Mitral	Unknown	None known	Valve replacement	Cure
Fukumoto [34]	2000	48	M	Japan	*multocida*	Unknown	None	Mitral	Dog	None known	Valve replacement	Cure
Fayad [35]	2001	48	M	France	*multocida*	Unknown	None	Aortic	Dog	Erysipelas, leg	Valve replacement	Coagulase-negative *Staphylococcus* endocarditis, cured after 1 month of antibiotics
Rosenbach [36]	2001	78	M	USA	*dagmatis*	Unknown	None	Aortic (prosthesis)	None	None known	None	Cure
Camou [18]	2003	81	F	France	*multocida*	Unknown	*Burkholderia cepaciae*, *Candida* sp.	Aortic	Cat	Thigh abscess	None	Death from septic shock
Sauvet [37]	2004	78	F	France	*multocida*	Unknown	None	Aortic	Unknown	None known	None	Heart failure with reduced ejection fraction; valve replacement planned for persistent vegetation
Al-Ghonaim [38]	2004	50	M	Saudi Arabia	*multocida*	Unknown	None	Aortic	Sheep	None known	Valve replacement	Cure
Graf [39]	2005	36	M	Austria	*multocida*	Unknown	None	Pulmonary	Cat, dog	None known	Valve replacement	Cure
Dan [40]	2005	43	?	Romania	*pneumotropica*	Unknown	None	Mitral	“Pets”	Scratched	Valve replacement	Cure
Reinsch [41]	2008	66	M	Germany	*multocida*	Unknown	None	Aortic (prosthesis)	Cat	Bite, leg	Valve replacement	*P aeruginosa* aortic valve endocarditis following dental work 4 months after discharge, leading to death
Naba [42]	2008	88	F	Lebanon	*multocida*	Unknown	None	Tricuspid	Cat	Bit, leg	None	Cure
Khan [43]	2011	82	M	USA	*multocida*	Unknown	None	Aortic	Cat	None known	None	Cure
Strahm [5]	2011	77	M	USA	*dagmatis*	Unknown	None	Aortic (prosthesis)	Cat	Licked pruritic rash	Valve replacement	Cure
Tirmizi [44]	2011	34	M	USA	*pneumotropica*	Unknown	None	Tricuspid	Rodents, fish	None known	None	Cure
Satta [45]	2012	38	M	UK	*multocida*	Unknown	None	Aortic	Cat, dog	None known	None	Cure
Fayad [46]	2012	62	M	France	*haemolytica*	Unknown	None	Aortic	Unknown	None known	Valve replacement	Cure
Mikaberidz [19]	2013	60	F	USA	*multocida*	Unknown	None	Aortic	Dog, cat	Sacral ulcer	Valve replacement	Cure
Yuji [47]	2015	50	M	Japan	*multocida*	Unknown	None	Mitral	None	None known	Valve replacement	Cure
Branch [48]	2015	50	M	Japan	*multocida*	Piperacillin-tazobactam	None	Mitral	None	None known	Valve replacement	Cure
Ahlsson [49]	2016	70	M	Sweden	*multocida*	Unknown	None	Aortic	Cat	Bites	Valve replacement	Cure
Porter	2019	66	F	USA	*multocida*	Unknown	*Staphylococcus epidermidis*, *Staphylococcus warneri*, *Curtobacterium* sp., *Klebsiella aerogenes*	Aortic (prosthesis), tricuspid	Cat	None known	Valve replacement	Cure

## Data Availability

The data used in our statistical analysis are a compilation of case data from *Pasteurella* endocarditis cases available in the peer-reviewed literature as cited in this manuscript and consist of patient characteristics, their treatment courses and clinical outcomes, and available information on the infecting bacterial species' characteristics. They are available by request from the corresponding author.
